# Pulsed Field Versus Cryoballoon Ablation for Atrial Fibrillation: Procedural Outcomes and Hemolysis-Related Laboratory Changes in a Single-Center Pilot Study

**DOI:** 10.3390/medicina62081580

**Published:** 2026-08-18

**Authors:** Kyunyeon Kim, YouMi Hwang, Youngjun Son, Jaehoon Kim, Sumin Roh

**Affiliations:** 1 Department of Cardiology, St. Vincent’s Hospital, The Catholic University of Korea, Suwon 16247, Republic of Korea; gemini729@naver.com (K.K.);; 2 Catholic Research Institute for Intractable Cardiovascular Disease (CRID), College of Medicine, The Catholic University of Korea, Seoul 06591, Republic of Korea

**Keywords:** atrial fibrillation, pulsed field ablation, cryoballoon ablation, hemolysis, acute kidney injury

## Abstract

*Background and Objectives*: Pulsed field ablation (PFA) and cryoballoon ablation (CRYO) are established approaches for pulmonary vein isolation in atrial fibrillation (AF). Given emerging concerns regarding hemolysis following PFA, this pilot study aims to directly compare outcomes between these modalities. This study aims to evaluate differences in laboratory parameters potentially compatible with hemolysis and procedural safety between PFA and CRYO among AF patients. *Materials and Methods*: In this retrospective single-center analysis, 33 symptomatic AF patients underwent either PFA (*n* = 17) or CRYO (*n* = 16). Hemoglobin, hematocrit, platelet count, total bilirubin and renal function were measured at all three pre-specified time points: baseline, immediately after ablation and on day 1. Group × time interactions were tested with linear mixed-effects models, and changes are reported with 95% confidence intervals (CIs). Acute kidney injury (AKI) was defined by KDIGO serum creatinine criteria and assessed individually; sensitivity analyses were restricted to pulmonary-vein-isolation (PVI)-only procedures. Additionally, procedural metrics and complication frequencies were analyzed between groups. *Results*: The baseline demographics and clinical characteristics did not differ significantly between both groups. Both ablation techniques led to substantial reductions in hemoglobin following the procedure, with no between-group difference (day-1 change −1.84 vs. −1.57 g/dL; difference −0.27, 95% CI −1.01 to +0.47; group × time *p* = 0.192). Notably, total bilirubin increased to a greater extent with PFA (day-1 change +0.48 vs. +0.15 mg/dL; difference +0.33, 95% CI +0.06 to +0.59; group × time *p* = 0.003), with the largest separation immediately after ablation (1.43 ± 0.36 vs. 0.99 ± 0.35 mg/dL, *p* = 0.001). No patient in either group met KDIGO criteria for AKI of any stage; the largest post-procedural rise in creatinine was +0.10 mg/dL in both groups. *Conclusions*: Both PFA and CRYO caused acute declines in hemoglobin, and total bilirubin rose more after PFA, with no accompanying change in renal function. Further studies involving larger cohorts and direct hemolysis biomarkers are necessary to corroborate these findings.

## 1. Introduction

Atrial fibrillation (AF) is recognized as the most prevalent cardiac arrhythmia, with catheter ablation now serving as a well-established rhythm-control intervention for patients experiencing symptoms [1]. Pulmonary vein isolation (PVI) remains the foundation of ablation approaches, and is typically achieved through radiofrequency (RF) ablation or cryoballoon ablation (CRYO) [2]. Both ablation techniques rely on thermal energy, which by nature lacks selectivity and can result in unintended injury to surrounding anatomical structures, including the esophagus, phrenic nerve, and pulmonary veins [3,4].

Pulsed field ablation (PFA) has recently emerged as a minimal-thermal alternative modality, utilizing irreversible electroporation to facilitate tissue selectivity while minimizing collateral injury [5]. Preclinical and early clinical studies have validated its efficacy and safety, demonstrating durable lesion formation and limited extracardiac damage [6,7,8,9,10,11]. Procedural similarities between PFA and CRYO, such as relatively short operation times and user-friendly workflows compared to point-by-point RF ablation, support their appeal as alternatives for effective PVI delivery. However, there remains a lack of sufficient direct clinical data comparing hemolysis markers and peri-procedural safety profiles between PFA and CRYO [12]. Erythrocyte injury associated with electroporation has been documented in preclinical research, and isolated cases of hemolysis-related acute kidney injury (AKI) have been reported within large PFA registries [13,14,15]. Despite the increasing clinical implementation of PFA, comparative analyses of hemolysis biomarker variations between PFA and CRYO are currently unavailable.

Accordingly, this study was designed to preliminarily compare the VARIPULSE PFA system (Biosense Webster, Irvine, CA, USA) and CRYO, with a specific focus on the dynamics of laboratory parameters potentially compatible with hemolysis and peri-procedural safety [16]. Through the assessment of laboratory parameters alongside procedural outcomes, the goal is to assess whether PFA demonstrates a safety profile comparable to CRYO, addressing both procedural and hemolytic risk, while retaining the practical benefits of a non-point-by-point ablation technique.

## 2. Methods

### 2.1. Study Design and Population

This retrospective single-center observational study was conducted from February 2025 to August 2025 at St. Vincent’s Hospital, The Catholic University of Korea. Eligible participants included symptomatic patients with AF who had not responded to at least one antiarrhythmic medication. The criteria for inclusion were as follows: (1) age ≥18 years, (2) paroxysmal or persistent AF, and (3) a clinical indication for catheter ablation. Exclusion criteria were significant structural heart disease—defined as moderate or severe valvular disease, hypertrophic or infiltrative cardiomyopathy, congenital heart disease, or a prosthetic valve—severe hepatic or hematologic disorders, and contraindications to anticoagulation. In total, 33 patients were recruited for the study. Of these, 17 patients underwent PFA using the VARIPULSE system, while 16 patients received CRYO. Assignment to either PFA or CRYO was based on operator availability and patient choice, rather than through randomization. Baseline demographic, clinical, echocardiographic, and laboratory characteristics were collected and entered into a dedicated institutional database. Prior to ablation, all patients underwent cardiac computed tomography (CT) scans to evaluate the pulmonary veins and surrounding anatomical structures. The study protocol received approval from the Institutional Review Board of St. Vincent’s Hospital and was conducted in accordance with the Declaration of Helsinki (Approval No.VC25RISI0183, approved 28 August 2025).

### 2.2. Ablation Procedure

All ablation procedures were conducted under local anesthesia with 1% lidocaine and conscious sedation. Analgosedation was provided using a combination of remifentanil, dexmedetomidine, and midazolam, with lighter sedation generally applied in CRYO cases and deeper sedation in PFA cases. Vascular access was achieved through both the right and left femoral veins using standard percutaneous puncture methods. Intracardiac echocardiography (ICE) was utilized to ensure procedural safety with either a 9 Fr ViewFlex ICE catheter (Abbott, Abbott Park, MN, USA) or a CartoSound catheter (Biosense Webster, Irvine, CA, USA). Heparin was administered prior to and throughout the procedure to maintain an activated clotting time (ACT) ≥ 300 s. Transseptal puncture (TSP) was performed via the right femoral vein using either an RF-powered transseptal needle (Medwin, Seoul, South Korea) or a BRK-1 needle (Abbott, Abbott Park, MN, USA) inserted through a Fast-Cath SL1 sheath (Abbott, Abbott Park, MN, USA). The fossa ovalis was located using ICE, and successful access to the LA was confirmed prior to advancing the sheath and dilator into the left atrium (LA). No contrast was administered intra-procedurally in either group; catheter navigation relied on ICE, fluoroscopy and, in the PFA group, the CARTO 3 electroanatomical mapping system. Cavotricuspid isthmus (CTI) ablation was performed only in patients with typical atrial flutter documented before the procedure or induced during it, and bidirectional isthmus block was confirmed at the end of the procedure. Superior vena cava (SVC) isolation was performed at the operator’s discretion in the PFA group.

### 2.3. Pulsed Field Ablation

In the PFA group, PVI was performed utilizing the VARIPULSE PFA system, which incorporates a proprietary TRUPULSE Generator and CARTO 3 electroanatomical mapping system (Biosense Webster, Irvine, CA, USA). After TSP, the SL1 sheath was exchanged for an 8 Fr CARTO VIZIGO steerable sheath (Biosense Webster, Irvine, CA, USA). A 7.5 Fr circular multipolar catheter (VARIPULSE) was introduced into the LA. The catheter was positioned at each pulmonary vein (PV) antrum with both ICE and fluoroscopic guidance, and pulsed field energy was delivered to achieve isolation. Additional energy applications were performed at the operator’s discretion if initial PVI was incomplete. The number of applications delivered was recorded in the procedure report and tabulated separately for the LA and SVC.

### 2.4. Cryoballoon Ablation

In the CRYO group, PVI was accomplished with the CryoConsole system (Medtronic, Minneapolis, MN, USA) equipped with a 28 mm Arctic Front Advance cryoballoon catheter (Medtronic, Minneapolis, MN, USA). After TSP, the SL1 sheath was exchanged for a dedicated steerable delivery sheath specifically designed for cryoballoon application. The cryoballoon catheter was then advanced to each pulmonary vein for isolation, with the freeze duration adjusted according to time to isolation (TTI) and nadir temperature. If TTI was ≤60 s, a 180 s freeze was conducted, whereas longer freeze durations (up to 240 s) were delivered when TTI exceeded 60 s. During right-sided PV ablation, continuous phrenic nerve pacing was utilized by positioning a catheter in the superior vena cava to monitor diaphragmatic motion and reduce the risk of phrenic nerve injury. Additional applications were administered at the operator’s discretion in the presence of incomplete PVI or PV reconnection.

### 2.5. Blood Sampling and Biochemistry Marker Analysis

Venous blood samples were collected at three predefined time points: within 24 h prior to the procedure (baseline), immediately after ablation, and 24 h post-procedure. Baseline and 24 h samples were drawn from an antecubital vein, whereas immediate post-procedure samples were collected through the femoral sheath during ablation. Complete blood counts were determined from EDTA-anticoagulated samples using automated hematology analyzers (XR-10, XR-30, XN-10, and XN-20; Sysmex Corporation, Kobe, Japan). Evaluation of biochemical parameters—including serum creatinine, blood urea nitrogen (BUN), and total/direct bilirubin—was conducted using plasma obtained from lithium heparin tubes in the central laboratory, and analyzed on an AU 5800 (Beckman Coulter, Brea, CA, USA) automated chemistry platform following standardized procedures. The estimated glomerular filtration rate (eGFR) was derived using the Chronic Kidney Disease Epidemiology Collaboration (CKD-EPI) equation [17]. All tests were conducted promptly after sample collection to ensure result accuracy. Acute kidney injury was defined a priori by the KDIGO 2012 serum creatinine criteria: an absolute increase of ≥0.3 mg/dL within 48 h or an increase to ≥1.5 times baseline, with every patient evaluated individually against both thresholds; the urine-output criterion could not be applied because hourly urine output was not recorded [18].

### 2.6. Statistical Analysis

Continuous variables are expressed as the mean ± standard deviation (SD) for normally distributed data, or as the median (interquartile range [IQR]) according to their distribution, with normality assessed by the Shapiro–Wilk test. Categorical variables are described by absolute counts and percentages. Comparisons between the PFA and CRYO groups were made with either the Welch t-test or Mann–Whitney U test for continuous data, and the χ^2^ test or Fisher’s exact test for categorical data. Longitudinal data were analyzed with linear mixed-effects models including a random intercept per patient and fixed effects for group, time (three levels) and the group × time interaction, tested by a joint Wald test; this model, rather than separate within-group paired tests, forms the basis of all between-group inference. Within-group changes and between-group differences in change are reported with 95% confidence intervals, and models for right-skewed variables were repeated on the logarithmic scale. Pre-specified sensitivity analyses were restricted to PVI-only lesion sets, adjusted for baseline value, SVC ablation and heparin dose by analysis of covariance, and repeated excluding the patient with an access-site complication; within the PFA group, the association between application number and biomarker change was assessed by Pearson and Spearman correlation. No adjustment was made for multiple comparisons across the thirteen parameters examined, and the achieved power for the day-1 bilirubin change was approximately 68% (observed d = 0.87). Statistical significance was set at a two-sided *p*-value < 0.05. Analyses were performed using SPSS Statistics, version 29.0 (IBM Corp., Armonk, NY, USA).

## 3. Results

### 3.1. Baseline Characteristics

Baseline characteristics of the study cohort are reported in Table 1. Mean age was similar between the PFA and CRYO groups (59.9 ± 7.9 vs. 61.2 ± 10.2 years, *p* = 0.683), and there was no significant difference in sex distribution (82.4% vs. 75.0%, *p* = 0.688). Anthropometric measures—including weight, height, and body mass index—were not significantly different between groups. The distribution of AF type was comparable, with most patients in each group presenting with persistent AF (64.7% vs. 50.0%, *p* = 0.491). The prevalence of cardiovascular comorbidities, such as hypertension, diabetes mellitus, coronary artery disease, heart failure, and obstructive sleep apnea, was well matched between the groups. Pre-procedural cardiac imaging, including cardiac CT and transthoracic echocardiography, demonstrated similar left atrial volume, left ventricular ejection fraction (LVEF), left ventricular end-diastolic diameter (LVEDd), E/e′ ratio, and left atrial volume index (LAVI) between cohorts.

### 3.2. Procedural Characteristics

Procedural characteristics are detailed in Table 2. Total procedure time showed no significant difference between groups (85.4 ± 15.5 vs. 90.6 ± 28.1 min, *p* = 0.511). The time from transseptal puncture (TSP) to procedure completion was likewise similar (59.6 ± 11.1 vs. 68.2 ± 19.4 min, *p* = 0.126). PVI was successful in all patients across both groups. SVC ablation was frequently performed in the PFA group (64.7% vs. 0%, *p* < 0.001). Conversely, CTI ablation was more commonly conducted in the CRYO group (25.0% vs. 5.9%, *p* = 0.175). Heparin dosage and maximum ACT did not differ significantly between groups. One patient in the PFA group experienced an extraperitoneal hematoma, with no complications reported in the CRYO group (*p* = 1.000).

### 3.3. Comparison Between PFA and CRYO Patients

**Figure 1:**
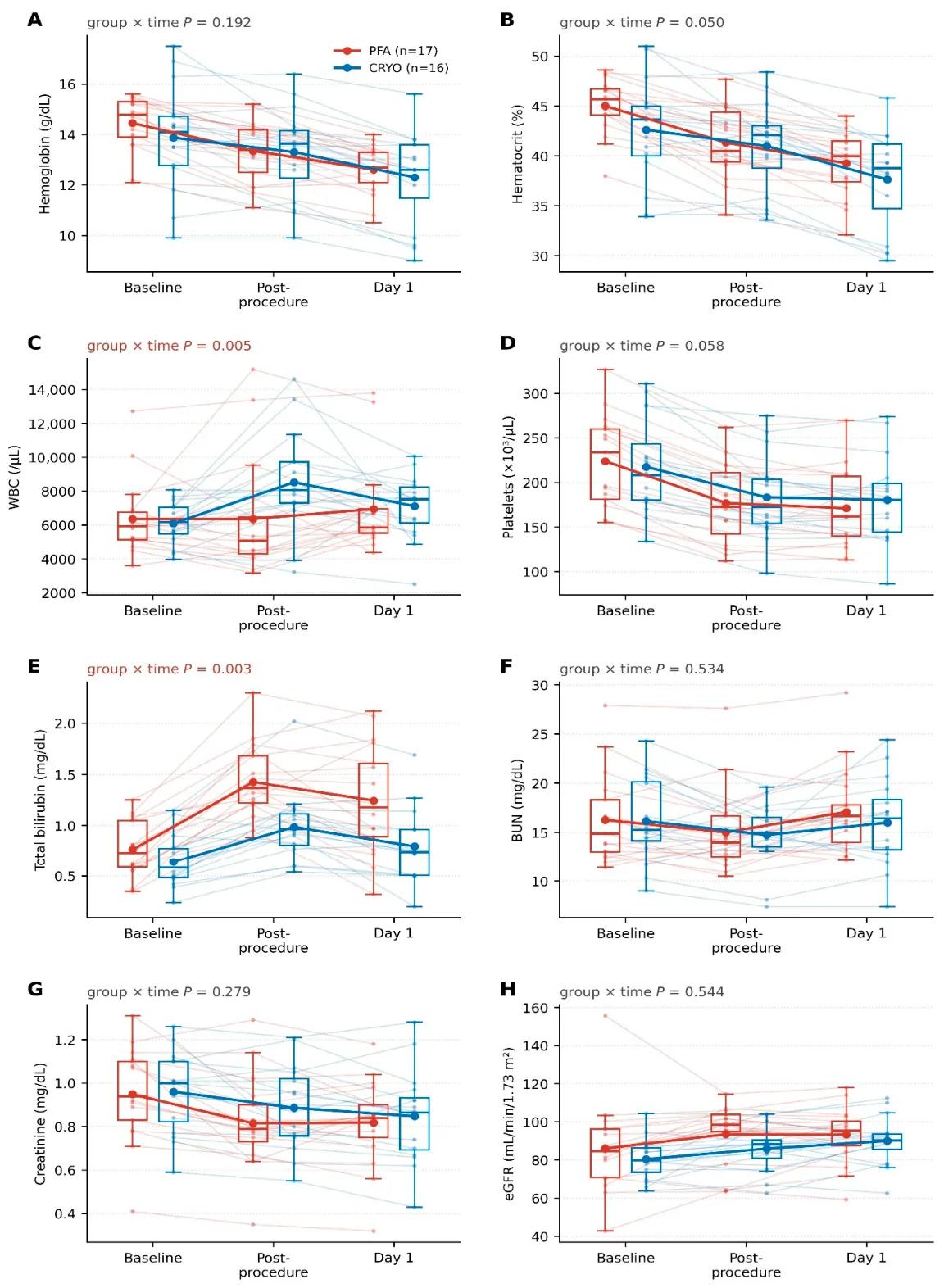
Boxplots show biomarker distributions at baseline, immediately after ablation and on the day after ablation (Day 1) in the PFA and CRYO groups. Panels: (**A**) hemoglobin; (**B**) hematocrit; (**C**) white blood cell count; (**D**) platelet count; (**E**) total bilirubin; (**F**) blood urea nitrogen; (**G**) creatinine; (**H**) eGFR. Center line = median; box = interquartile range (IQR); whiskers = 1.5 × IQR; faint lines connect the three measurements of each individual patient, and all data points are shown, with no observations excluded; the bold line with markers shows the group mean trajectory. The exact *p*-value for the group × time interaction from the linear mixed-effects model is printed on each panel and highlighted where *p* < 0.05. CRYO = cryoballoon ablation; eGFR = estimated glomerular filtration rate; IQR = interquartile range; PFA = pulsed field ablation; WBC = white blood cell count.

Baseline laboratory parameters were generally similar between the PFA and CRYO groups, with no significant differences identified in hemoglobin (Hb), hematocrit, white blood cell count (WBC), platelet count (PLT), renal function markers [BUN, creatinine (Cr), eGFR], cardiac enzymes [creatine kinase (CPK), creatine kinase-MB (CK-MB)], or bilirubin levels (Table 3). Values at all three sampling points are reported in Table 3 with the exact group × time interaction *p*-value, and displayed as paired individual trajectories in Figure 1.

On the day following ablation, significant reductions in Hb, hematocrit, and PLT were observed in both groups compared to baseline. However, the degree of these changes did not differ significantly between groups (day-1 change in Hb −1.84 vs. −1.57 g/dL; difference −0.27, 95% CI −1.01 to +0.47, *p* = 0.458; group × time *p* = 0.192) (Table 4). Importantly, total bilirubin responses diverged between cohorts. In the PFA group, bilirubin increased markedly from 0.76 ± 0.27 to 1.24 ± 0.53 mg/dL (*p* < 0.001), while the CRYO group experienced only a mild, statistically nonsignificant rise (0.64 ± 0.25 to 0.80 ± 0.36 mg/dL; *p* = 0.087). The between-group difference in change was +0.33 mg/dL (95% CI +0.06 to +0.59, *p* = 0.018), with a group × time interaction of *p* = 0.003; on the logarithmic scale, the interaction was not significant (*p* = 0.109). The separation was largest in the sample taken immediately after ablation (1.43 ± 0.36 vs. 0.99 ± 0.35 mg/dL, *p* = 0.001) and had partially resolved by day 1 (*p* = 0.008). No patient in either group exceeded twice the upper limit of normal at any time point. Both groups demonstrated substantial elevations in CPK and CK-MB values from baseline to day 1, without significant group differences at day 1, although the kinetics differed markedly, with an early peak after CRYO and a delayed rise after PFA (both interaction *p* < 0.001). While WBC counts tended to increase in both groups, these changes did not reach statistical significance in the PFA group (interaction *p* = 0.005). Renal function remained unaffected, as shown by stable Cr and eGFR measurements in both groups.

### 3.4. Sensitivity Analyses and Renal Safety

**Figure 2:**
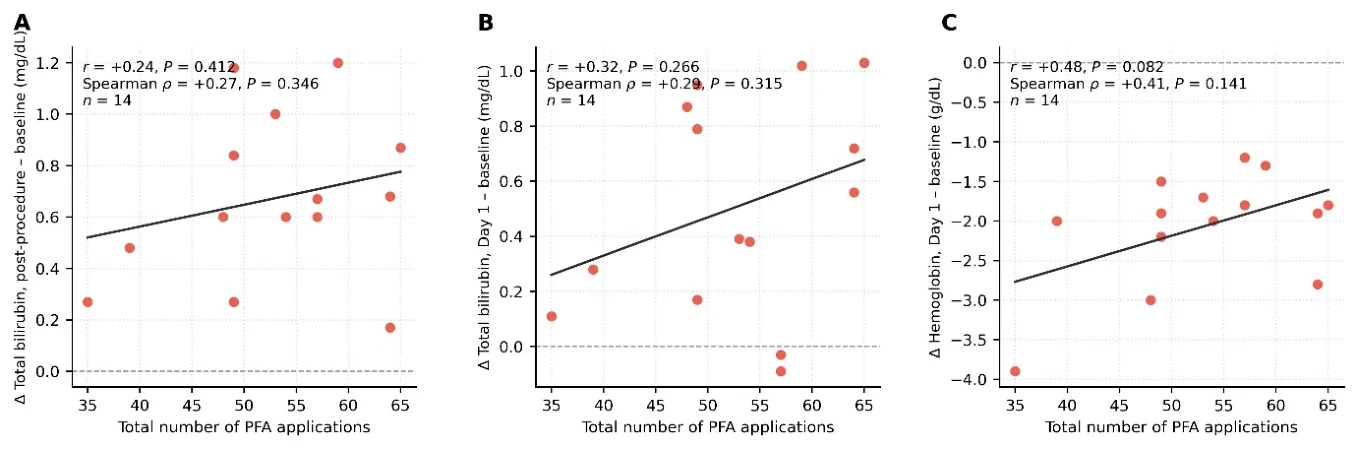
Relationship between the number of pulsed field applications and change in laboratory parameters. (**A**) Change in total bilirubin from baseline to immediately after ablation; (**B**) change in total bilirubin from baseline to day 1; (**C**) change in hemoglobin from baseline to day 1. Each point is one PFA patient (*n* = 14 with retrievable application counts); the solid line is the least-squares fit. PFA = pulsed field ablation.

Because the lesion sets differed, all analyses were repeated in the 17 patients whose ablation was confined to PVI (5 PFA, 12 CRYO; Table 5). The direction of the bilirubin difference was preserved but the confidence interval included no difference (day-1 change +0.45 ± 0.49 vs. +0.14 ± 0.38 mg/dL; difference +0.31, 95% CI −0.28 to +0.90, *p* = 0.248), and the group × time interaction in this subgroup was *p* = 0.179. Within the PFA group, patients who did and did not undergo SVC ablation showed almost identical increases (+0.50 ± 0.41 vs. +0.45 ± 0.43 mg/dL, *p* = 0.805). In an analysis of covariance adjusted for baseline bilirubin, the PFA–CRYO difference was +0.33 mg/dL (95% CI +0.05 to +0.61, *p* = 0.023), and after further adjustment for SVC ablation and heparin dose it was +0.31 mg/dL (95% CI −0.09 to +0.72, *p* = 0.123). Excluding the patient with an access-site complication left the estimates unchanged (+0.33 mg/dL, 95% CI +0.05 to +0.61, *p* = 0.021). Correlations between the number of PFA applications and biomarker change were positive but not significant (day-1 Hb r = +0.48, *p* = 0.082; day-1 bilirubin r = +0.32, *p* = 0.266; Table 6 and Figure 2).

**Figure 3:**
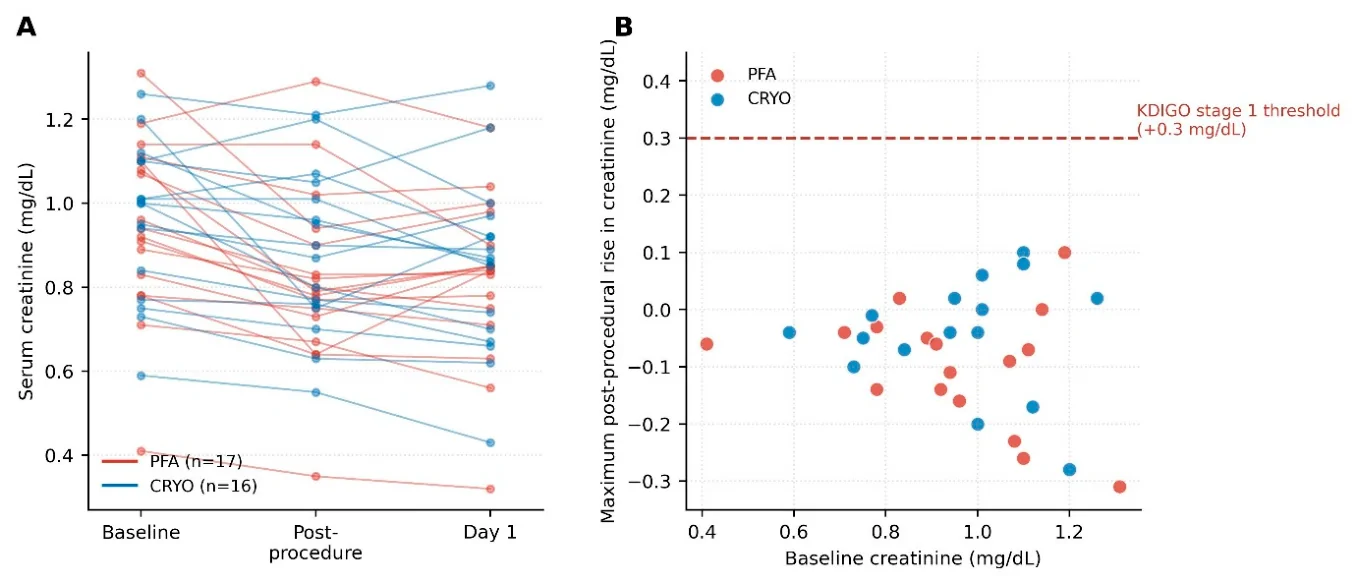
Individual-level assessment of renal safety. (**A**) Serum creatinine trajectories for every patient across the three time points, by group. (**B**) Maximum post-procedural rise in serum creatinine plotted against the baseline value; the dashed line marks the KDIGO stage-1 threshold of +0.3 mg/dL. No patient crossed this threshold. CRYO = cryoballoon ablation; KDIGO = Kidney Disease: Improving Global Outcomes; PFA = pulsed field ablation.

No patient in either group met the KDIGO criteria for AKI of any stage (Table 7 and Figure 3). The largest absolute post-procedural rise in creatinine was +0.10 mg/dL and the largest relative rise was 1.09 times baseline in both groups; no patient received transfusion or renal replacement therapy. The single extraperitoneal hematoma was managed conservatively, and that patient’s hemoglobin did not fall (12.1 to 12.5 g/dL). With 17 and 16 patients the upper 95% bounds for an unobserved event were 17.6% and 18.8%, so these data cannot establish equivalent safety.

## 4. Discussion

In this retrospective, single-center comparison of non-point-by-point AF ablation techniques, we observed that both PFA employing a multielectrode circular system and CRYO ablation were completed safely, with successful PVI achieved in all patients and no episodes of AKI. Procedure durations were comparable for the two groups, while the PFA group experienced significantly lower fluoroscopy times (Table 2). SVC ablation was commonly conducted in the PFA arm, reflecting the deliberate omission of SVC ablation during CRYO due to the increased risk of complications such as phrenic nerve palsy [19]. Both groups exhibited laboratory changes potentially compatible with mild, transient hemolysis. Of note, post-procedural bilirubin increases were more pronounced with PFA than CRYO, while serum creatinine and eGFR did not change significantly in either group. One case of extraperitoneal access-site hematoma occurred in the PFA group; no other major periprocedural complications were reported.

Two principal findings should be highlighted. First, no patient in either group met KDIGO criteria for AKI and complication rates remained low, although with 17 and 16 patients these data cannot establish that the platforms are equally safe. Second, PFA was linked to a more pronounced increase in bilirubin by day 1. However, changes in hemoglobin were similar between the groups, and the bilirubin difference was not confirmed after logarithmic transformation or in the PVI-only subgroup, so an effect of lesion set rather than of the energy source cannot be excluded. These observations are consistent with, but do not demonstrate, subclinical intravascular hemolysis at the application counts employed in this study.

### 4.1. Context Within Prior Study and Potential Mechanisms

Our findings are consistent with recent studies showing that PFA more frequently leads to laboratory markers of hemolysis compared to thermal ablation, and that the degree of hemolysis correlates with the number of pulsed-field applications [14,20,21,22,23]. Previous studies have reported increased levels of free hemoglobin and reduced haptoglobin, as well as a higher incidence of hemoglobinuria after PFA compared to radiofrequency ablation [14,20,23]. However, clinically significant anemia is uncommon, and AKI appears rare; when reported, these outcomes are generally observed with high pulse counts or insufficient hydration [15,24]. Taken together, these results, along with our finding of increased bilirubin in the absence of creatinine elevation, support the notion that PFA-induced hemolysis is generally mild and clinically inapparent but should be recognized and addressed in procedural protocols.

Hemolysis following PFA is biologically plausible, as high-amplitude electric fields can induce the formation of nanoscale pores in erythrocyte membranes (irreversible electroporation), which subsequently result in osmotic swelling and cell lysis [25]. This process is mediated by the energy field and is anticipated to correlate with local field strength, the degree of catheter–tissue coupling, and the total number of applications [26]. A recent narrative review synthesizes this evidence and identifies peak output voltage, catheter–tissue contact quality and, above all, the number of applications as the determinants of hemolysis burden, with the probability of AKI rising particularly once applications exceed approximately 90–100, and with pre-existing renal dysfunction lowering that threshold [27]. Both observations bear directly on our cohort, in which the median application count was 54 (range 35–65) and baseline renal function was preserved. The greater increase in bilirubin observed with PFA in our cohort, without an accompanying elevation in creatinine, is compatible with limited intravascular hemolysis that is efficiently cleared by the haptoglobin–hemopexin system before the onset of renal injury [13,24]. In our study, the number of applications was modest, with most lesions limited to the LA and SVC, which may account for the lack of AKI [15,21]. Additionally, in vitro research has shown that hemolysis is exacerbated by suboptimal catheter-tissue contact, which increases the exposure of circulating blood to the electric field [28].

### 4.2. Clinical Implications

From a practical standpoint, these findings have important implications for current clinical practice. Under the currently recommended energy settings and with standard periprocedural hydration, both PFA and CRYO produced no detectable renal injury in our cohort. Given the more pronounced post-PFA increase in bilirubin, it may be advisable to routinely obtain a day-1 chemistry panel that includes bilirubin, particularly in patients with baseline renal impairment or when extensive non-PV lesions are anticipated. Considering previously reported dose–response relationships [27,28], limiting the total number of PFA applications and ensuring optimal catheter–tissue contact may help decrease hemolysis risk without sacrificing effectiveness; protocolized rather than discretionary hydration should be adopted to lower the chance of renal injury in patients at increased risk [15], a recommendation our own inability to quantify administered fluid volumes illustrates the need for. Lastly, the notably shorter fluoroscopy times observed in our study highlight a workflow efficiency advantage of PFA when used with an integrated 3D mapping platform. This result contrasts with previous meta-analyses reporting longer fluoroscopy times for 3D mapping systems. The observed differences from meta-analyses that found longer fluoroscopy times for PFA probably reflect variations in procedural workflows [6]. The integration of the VARIPULSE system with CARTO 3 facilitates minimal- or zero-fluoroscopy ablation, as demonstrated by the AdmIRE trial (median fluoroscopy time: 7.1 min; 25.3% of cases conducted without fluoroscopy) [29], thereby minimizing radiation exposure for patients and operators.

### 4.3. Limitations and Future Directions

Several limitations should be considered when interpreting these results. First, and most importantly, hemolysis was not directly demonstrated. Haptoglobin, plasma-free hemoglobin, lactate dehydrogenase, reticulocyte count and urinalysis for hemoglobinuria were not measured, and post-procedural bilirubin was not fractionated [14,20,22]. The observed hemoglobin reduction and bilirubin elevation may also reflect procedural blood loss, phlebotomy, hemodilution, fasting, deeper sedation, or transient hepatic congestion. Accordingly, these findings are described only as laboratory changes potentially compatible with hemolysis, which represents the principal limitation of this study. Second, only a single PFA platform (a multielectrode circular system with integrated 3D mapping) was utilized, which limits the generalizability of these findings to other PFA systems with varying pulse structures or unipolar setups [16]. Third, our cohort was small, conducted at a single center, and lacked randomization, thereby introducing possible selection and confounding biases (including a higher prevalence of SVC ablation in the PFA group and more frequent CTI ablation in the CRYO group). The PVI-only sensitivity analysis addressing the lesion-set imbalance included only 5 and 12 patients and was correspondingly imprecise. Importantly, as a pilot investigation, this analysis lacked sufficient statistical power to identify subtle inter-group differences or rare adverse outcomes, leading to the possibility of type II errors for findings that did not achieve statistical significance. The study was not powered to detect infrequent safety outcomes such as AKI, nor did it evaluate long-term renal function, and the urine-output criterion for AKI could not be applied. Finally, peri-procedural hydration volumes were not standardized or recorded, a factor that could influence both hemolysis kinetics and renal elimination.

Larger, randomized, multicenter studies directly comparing current PFA and CRYO protocols should include measurement of direct hemolysis biomarkers collected immediately after ablation and at sequential time points, bilirubin fractionation at every time point, standardized hydration strategies, and systematic lesion application tracking within both PV-only and extended ablation sets. Such research may establish exposure thresholds for clinically significant hemolysis, elucidate device-specific profiles, and determine whether protocolized hydration management and procedural oversight result in improved renal outcomes. The addition of ICE-guided contact assessment and mapping feedback could further support optimization of energy delivery and a reduction in unintended effects. Despite these study limitations, our results offer preliminary evidence that laboratory changes potentially compatible with mild hemolysis after PFA are not accompanied by clinically apparent renal injury at the application counts used here, supporting ongoing evaluation in the broader clinical setting.

## 5. Conclusions

In this preliminary study, PFA appeared to offer procedural efficiency comparable to CRYO, and no patient in either group developed acute kidney injury. Post-procedural elevations in bilirubin were more pronounced after PFA and may be associated with limited intravascular hemolysis, although this could not be confirmed with direct biomarkers and no renal complications were observed. Given the retrospective design and the small sample, these findings should be regarded as hypothesis-generating; they suggest that PFA may represent a safe and biologically selective alternative to AF ablation, but larger prospective studies are required.

## Figures and Tables

**Table 1:** Baseline characteristics.

	PFA Group (*n* = 17)	CRYO Group (*n* = 16)	*p*-Value
Age (year)	59.88 ± 7.91	61.19 ± 10.17	0.683
Sex			
Female	3 (17.6%)	4 (25.0%)	0.688
Male	14 (82.4%)	12 (75.0%)	
Body weight (kg)	73.96 ± 12.79	73.93 ± 15.02	0.995
Height (cm)	168.50 ± 6.85	167.90 ± 6.58	0.799
BMI	25.91 ± 3.27	26.11 ± 4.47	0.883
AF type			
Paroxysmal	6 (35.3%)	8 (50.0%)	0.491
Persistent	11 (64.7%)	8 (50.0%)	
CHA2DS2-VASc score	1.00 (1.00–2.00)	2.00 (1.75–3.00)	0.051
Heart failure	4 (23.5%)	7 (43.8%)	0.282
Hypertension	7 (41.2%)	8 (50.0%)	0.732
DM	3 (17.6%)	1 (6.2%)	0.601
CAD	4 (23.5%)	2 (12.5%)	0.656
MI history	0 (0.0%)	1 (6.2%)	0.485
Stroke history	1 (5.9%)	2 (12.5%)	0.601
OSA	2 (11.8%)	1 (6.2%)	1.000
LA volume (sys, cm^3^)	109.60 ± 46.34	98.56 ± 35.63	0.451
LA volume (dia, cm^3^)	136.46 ± 39.63	126.47 ± 34.45	0.447
LVEF (%)	56.16 ± 8.30	57.99 ± 8.45	0.537
LVEDd (mm)	48.62 ± 4.09	50.56 ± 4.11	0.183
E/e’	8.89 ± 2.08	9.58 ± 3.02	0.444
LAVI (mL/m^2^)	40.96 ± 16.79	32.15 ± 12.35	0.098
Current anticoagulation	17 (100.0%)	16 (100.0%)	1.000
Current AAD use			
Amiodarone	14 (82.4%)	10 (62.5%)	0.259
Propafenone	3 (17.6%)	1 (6.2%)	0.601

Data are shown as mean ± SD, median (interquartile range) or *n* (%). Normality was assessed with the Shapiro–Wilk test; the CHA2DS2-VASc score was non-normally distributed and was compared with the Mann–Whitney U test. AAD = antiarrhythmic drug; AF = atrial fibrillation; BMI = body mass index; CAD = coronary artery disease; CRYO = cryoballoon ablation; DM = diabetes mellitus; IQR = interquartile range; LA = left atrium; LAVI = left atrial volume index; LVEDd = left ventricular end-diastolic dimension; LVEF = left ventricular ejection fraction; MI = myocardial infarction; OSA = obstructive sleep apnea; PFA = pulsed field ablation.

**Table 2:** Procedural characteristics.

	PFA Group (n = 17)	CRYO Group (n = 16)	*p*-Value
Procedure time (min)	85.41 ± 15.51	90.62 ± 28.14	0.511
Time after TSP (min)	59.59 ± 11.09	68.19 ± 19.43	0.126
Fluoroscopy time (min)	9.61 ± 3.30	18.04 ± 4.45	<0.001
Total exposure (Gycm^2^)	2.19 ± 1.04	3.24 ± 1.48	0.024
PFA total application	53.00 ± 8.09	n/a	n/a
PFA LA application	50.00 ± 7.71	n/a	n/a
PFA SVC application	3.00 ± 2.37	n/a	n/a
PVI	17 (100.0%)	16 (100.0%)	1.000
SVC ablation	11 (64.7%)	0 (0.0%)	<0.001
CTI ablation	1 (5.9%)	4 (25.0%)	0.175
Total heparin dose (IU)	8352.94 ± 1114.74	8687.50 ± 1537.04	0.478
maximum ACT (sec)	317.88 ± 36.55	330.25 ± 41.13	0.368
DCCV	11 (64.7%)	9 (56.2%)	0.728
Complication	1 (5.9%)	0 (0.0%)	1.000

Data are shown as mean ± SD or *n* (%). ACT = activated clotting time; CTI = cavotricuspid isthmus; CRYO = cryoballon ablation; DCCV = direct current cardioversion; LA = left atrium; n/a = not applicable; PFA = pulsed field ablation; PVI = pulmonary vein isolation; RA = right atrium; SVC = superior vena cava; TSP = transseptal puncture.

**Table 3:** Laboratory parameters at baseline, immediately after ablation and on day 1.

	Baseline			Immediately Post Procedure			Day 1			Group × Time
	PFA	CRYO	*p*	PFA	CRYO	*p*	PFA	CRYO	*p*	*p*
Hemoglobin (g/dL)	14.45 ± 1.10	13.88 ± 2.06	0.328	13.35 ± 1.20	13.31 ± 1.80	0.931	12.61 ± 1.01	12.31 ± 1.86	0.568	0.192
Hematocrit (%)	45.02 ± 2.79	42.62 ± 5.16	0.114	41.34 ± 3.54	40.99 ± 4.44	0.803	39.26 ± 3.32	37.64 ± 4.94	0.282	0.050
WBC (/µL)	6368 ± 2190	6101 ± 1233	0.667	6365 ± 3387	8528 ± 2941	0.059	6950 ± 2656	7123 ± 1893	0.830	0.005
MCV (fL)	93.19 ± 3.11	94.20 ± 6.51	0.578	93.18 ± 3.25	94.51 ± 6.16	0.450	93.39 ± 3.23	94.01 ± 6.03	0.719	0.193
MCH (pg)	29.88 ± 1.31	30.62 ± 2.72	0.333	30.07 ± 1.27	30.66 ± 2.59	0.423	30.02 ± 1.15	30.69 ± 2.62	0.358	0.596
MCHC (g/dL)	32.09 ± 1.14	32.45 ± 1.19	0.388	32.28 ± 0.94	32.39 ± 1.28	0.779	32.14 ± 0.91	32.62 ± 1.19	0.207	0.129
Platelets (×10^3^/µL)	223.94 ± 52.20	217.62 ± 53.54	0.734	177.00 ± 43.94	183.38 ± 47.21	0.691	171.12 ± 42.50	180.62 ± 49.40	0.559	0.058
Total bilirubin (mg/dL)	0.76 ± 0.27	0.64 ± 0.25	0.187	1.43 ± 0.36	0.99 ± 0.35	0.001	1.24 ± 0.53	0.80 ± 0.36	0.008	0.003
BUN (mg/dL)	16.28 ± 4.56	16.16 ± 4.27	0.941	15.02 ± 4.25	14.75 ± 3.34	0.841	17.09 ± 4.35	16.00 ± 4.48	0.483	0.534
Creatinine (mg/dL)	0.95 ± 0.21	0.96 ± 0.18	0.866	0.82 ± 0.21	0.89 ± 0.19	0.320	0.82 ± 0.20	0.85 ± 0.21	0.684	0.279
eGFR (mL/min/1.73 m^2^)	86.09 ± 23.66	80.48 ± 10.73	0.385	93.49 ± 16.00	86.00 ± 11.95	0.137	93.33 ± 14.66	90.05 ± 12.71	0.496	0.544
CPK (U/L)	147.76 ± 57.21	108.94 ± 61.97	0.072	168.41 ± 54.19	273.06 ± 96.35	<0.001	251.29 ± 63.48	256.00 ± 84.83	0.859	<0.001
CK-MB (ng/mL)	2.77 ± 2.76	1.65 ± 0.55	0.121	11.17 ± 7.81	35.76 ± 23.76	<0.001	24.38 ± 8.55	22.96 ± 9.39	0.654	<0.001

Data are mean ± SD. Between-group *p*-values at each time point are from Welch’s t-test. The final column gives the exact *p*-value for the group × time interaction from a linear mixed-effects model with a random intercept per patient and fixed effects for group, time (three levels) and their interaction, tested by a joint Wald test across both interaction terms. On the log scale, the group × time interaction for total bilirubin was *p* = 0.109. No adjustment was made for multiple comparisons. BUN = blood urea nitrogen; CK-MB = creatine kinase-MB; CPK = creatine phosphokinase; CRYO = cryoballoon ablation; eGFR = estimated glomerular filtration rate; MCH = mean corpuscular hemoglobin; MCHC = mean corpuscular hemoglobin concentration; MCV = mean corpuscular volume; PFA = pulsed field ablation; WBC = white blood cell count.

**Table 4:** Within-group changes and between-group differences in change, with 95% confidence intervals.

Parameter	Contrast	PFA: Change (95% CI) [*p*]	CRYO: Change (95% CI) [*p*]	Difference in Change (95% CI)	*p*
Hemoglobin (g/dL)	Baseline → post	−1.10 (−1.48 to −0.72) [<0.001]	−0.57 (−1.07 to −0.07) [0.029]	−0.53 (−1.14 to +0.08)	0.084
	Baseline → Day 1	−1.84 (−2.31 to −1.37) [<0.001]	−1.57 (−2.18 to −0.96) [<0.001]	−0.27 (−1.01 to +0.47)	0.458
Hematocrit (%)	Baseline → post	−3.68 (−4.81 to −2.54) [<0.001]	−1.64 (−3.13 to −0.15) [0.033]	−2.04 (−3.84 to −0.24)	0.028
	Baseline → Day 1	−5.75 (−7.03 to −4.48) [<0.001]	−4.98 (−6.72 to −3.24) [<0.001]	−0.77 (−2.85 to +1.31)	0.453
WBC (/µL)	Baseline → post	−3 (−1311 to +1305) [0.996]	+2427 (+1121 to +3732) [0.001]	−2430 (−4203 to −657)	0.009
	Baseline → Day 1	+582 (−454 to +1619) [0.251]	+1022 (+80 to +1965) [0.035]	−440 (−1785 to +904)	0.509
Platelets (×10^3^/µL)	Baseline → post	−46.94 (−54.68 to −39.20) [<0.001]	−34.25 (−46.75 to −21.75) [<0.001]	−12.69 (−26.91 to +1.53)	0.078
	Baseline → Day 1	−52.82 (−63.61 to −42.04) [<0.001]	−37.00 (−51.06 to −22.94) [<0.001]	−15.82 (−32.87 to +1.22)	0.068
Total bilirubin (mg/dL)	Baseline → post	+0.66 (+0.51 to +0.82) [<0.001]	+0.34 (+0.21 to +0.48) [<0.001]	+0.32 (+0.12 to +0.52)	0.003
	Baseline → Day 1	+0.48 (+0.27 to +0.69) [<0.001]	+0.15 (−0.03 to +0.34) [0.087]	+0.33 (+0.06 to +0.59)	0.018
BUN (mg/dL)	Baseline → post	−1.26 (−2.57 to +0.05) [0.058]	−1.41 (−2.95 to +0.12) [0.069]	+0.15 (−1.78 to +2.09)	0.872
	Baseline → Day 1	+0.82 (−0.38 to +2.01) [0.165]	−0.16 (−2.10 to +1.77) [0.860]	+0.98 (−1.22 to +3.18)	0.368
Creatinine (mg/dL)	Baseline → post	−0.13 (−0.20 to −0.06) [<0.001]	−0.07 (−0.14 to −0.01) [0.030]	−0.06 (−0.15 to +0.03)	0.200
	Baseline → Day 1	−0.13 (−0.18 to −0.08) [<0.001]	−0.11 (−0.17 to −0.06) [<0.001]	−0.02 (−0.09 to +0.05)	0.617
eGFR (mL/min/1.73 m^2^)	Baseline → post	+7.40 (−0.73 to +15.53) [0.072]	+5.52 (+0.07 to +10.98) [0.048]	+1.87 (−7.58 to +11.33)	0.688
	Baseline → Day 1	+7.24 (+0.19 to +14.28) [0.045]	+9.57 (+4.89 to +14.26) [<0.001]	−2.33 (−10.50 to +5.83)	0.563
CPK (U/L)	Baseline → post	+20.65 (−7.49 to +48.78) [0.139]	+164.12 (+100.49 to +227.76) [<0.001]	−143.48 (−211.47 to −75.49)	<0.001
	Baseline → Day 1	+103.53 (+64.46 to +142.59) [<0.001]	+147.06 (+98.71 to +195.41) [<0.001]	−43.53 (−103.28 to +16.21)	0.147
CK-MB (ng/mL)	Baseline → post	+8.41 (+4.44 to +12.37) [<0.001]	+34.10 (+21.42 to +46.78) [<0.001]	−25.70 (−38.80 to −12.59)	<0.001
	Baseline → Day 1	+21.62 (+17.17 to +26.07) [<0.001]	+21.31 (+16.34 to +26.28) [<0.001]	+0.31 (−6.10 to +6.71)	0.923

“Post” denotes the sample obtained immediately after ablation. Within-group changes are mean change with 95% confidence interval, with the *p*-value from a one-sample t-test in square brackets. The difference in change is the PFA-minus-CRYO difference with its 95% confidence interval, from Welch’s t-test. A significant within-group change in one arm and a non-significant change in the other does not by itself indicate a between-group difference; inference should be based on the difference in change and on the group × time interaction reported in Table 3. BUN = blood urea nitrogen; CI = confidence interval; CK-MB = creatine kinase-MB; CPK = creatine phosphokinase; CRYO = cryoballoon ablation; eGFR = estimated glomerular filtration rate; PFA = pulsed field ablation; WBC = white blood cell count.

**Table 5:** Sensitivity analysis restricted to pulmonary-vein-isolation-only procedures.

	PFA (*n* = 5)	CRYO (*n* = 12)	Difference (95% CI)	*p*
Δ Total bilirubin, baseline → post (mg/dL)	+0.45 ± 0.27	+0.35 ± 0.30	+0.10 (−0.24 to +0.44)	0.514
Δ Total bilirubin, baseline → Day 1 (mg/dL)	+0.45 ± 0.49	+0.14 ± 0.38	+0.31 (−0.28 to +0.90)	0.248
Total bilirubin, post procedure (mg/dL)	1.33 ± 0.36	1.01 ± 0.37	+0.32 (−0.12 to +0.77)	0.133
Total bilirubin, day 1 (mg/dL)	1.33 ± 0.67	0.79 ± 0.39	+0.53 (−0.28 to +1.35)	0.155
Δ Hemoglobin, baseline → day 1 (g/dL)	−2.50 ± 0.92	−1.60 ± 1.17	−0.90 (−2.10 to +0.30)	0.124
Δ Hematocrit, baseline → day 1 (%)	−7.78 ± 2.70	−5.10 ± 3.24	−2.68 (−6.13 to +0.77)	0.113
Δ Creatinine, baseline → day 1 (mg/dL)	−0.17 ± 0.13	−0.10 ± 0.11	−0.07 (−0.23 to +0.09)	0.339

Restricted to patients in whom neither superior vena cava nor cavotricuspid isthmus ablation was performed. Data are mean ± SD; differences are PFA minus CRYO with 95% confidence intervals from Welch’s t-test. In a mixed-effects model restricted to this subgroup, the group × time interaction for total bilirubin was not significant (*p* = 0.179). The direction of the bilirubin difference is preserved but all confidence intervals include zero; this subgroup is small and the analysis cannot exclude confounding by lesion set. CI = confidence interval; CRYO = cryoballoon ablation; PFA = pulsed field ablation.

**Table 6:** Relationship between the number of pulsed field applications and change in laboratory parameters (PFA group, *n* = 14).

Change in Parameter	Pearson r	*p*	Spearman ρ	*p*	Applications
Δ Total bilirubin, baseline → post	+0.24	0.412	+0.27	0.346	Total
Δ Total bilirubin, baseline → day 1	+0.32	0.266	+0.29	0.315	Total
Δ Hemoglobin, baseline → day 1	+0.48	0.082	+0.41	0.141	Total
Δ Hematocrit, baseline → day 1	+0.40	0.156	+0.35	0.218	Total
Δ Creatinine, baseline → day 1	−0.05	0.877	−0.10	0.746	Total
Δ Total bilirubin, baseline → day 1	+0.37	0.189	+0.32	0.263	Left atrium
Δ Hemoglobin, baseline → day 1	+0.44	0.115	+0.38	0.184	Left atrium
Δ Total bilirubin, baseline → day 1	−0.13	0.669	−0.03	0.923	SVC
Δ Hemoglobin, baseline → day 1	+0.21	0.479	+0.15	0.609	SVC

Application counts were retrievable for 14 of 17 PFA patients (median 54, interquartile range 49–58, range 35–65). Positive correlations with the change in hemoglobin and bilirubin are of the expected direction but none reached statistical significance; with *n* = 14 and a narrow exposure range this analysis is markedly underpowered and should not be read as evidence against a dose–response relationship. See also Figure 2. PFA = pulsed field ablation; SVC = superior vena cava.

**Table 7:** Individual-level assessment of acute kidney injury and other safety endpoints.

	PFA (*n* = 17)	CRYO (*n* = 16)	*p*-Value
**Acute kidney injury (KDIGO creatinine criteria)**			
Any stage	0 (0.0%)	0 (0.0%)	1.000
Rise ≥ 0.3 mg/dL within 48 h	0 (0.0%)	0 (0.0%)	1.000
Rise ≥ 1.5 × baseline	0 (0.0%)	0 (0.0%)	1.000
Maximum absolute rise in creatinine (mg/dL)	+0.10	+0.10	—
Maximum relative rise (× baseline)	1.09	1.09	—
**Hyperbilirubinemia**			
Total bilirubin > 1.2 mg/dL, post procedure	13 (76.5%)	2 (12.5%)	<0.001
Total bilirubin > 1.2 mg/dL, day 1	8 (47.1%)	2 (12.5%)	0.056
Total bilirubin > 2.4 mg/dL at any point	0 (0.0%)	0 (0.0%)	1.000
Peak total bilirubin observed (mg/dL)	2.30	2.02	—
**Anemia and bleeding**			
Hemoglobin fall ≥ 2 g/dL by day 1	6 (35.3%)	4 (25.0%)	0.712
Red-cell transfusion	0 (0.0%)	0 (0.0%)	1.000
Access-site complication	1 (5.9%)	0 (0.0%)	1.000
Renal replacement therapy	0 (0.0%)	0 (0.0%)	1.000

Acute kidney injury was defined a priori by the KDIGO 2012 serum creatinine criteria: an absolute increase of ≥0.3 mg/dL within 48 h or an increase to ≥ 1.5 times baseline. Each patient was evaluated individually against both thresholds. The urine-output criterion could not be applied because hourly urine output was not recorded. With 17 and 16 patients, the upper 95% confidence bounds for an event not observed were 17.6% and 18.8%, respectively; these data therefore cannot establish equivalence of safety between platforms. See also Figure 3. CRYO = cryoballoon ablation; KDIGO = Kidney Disease: Improving Global Outcomes; PFA = pulsed field ablation.

## Data Availability

The data presented in this study are available on request from the corresponding author due to privacy and ethical restrictions.
